# Sentinel lymph node detection in endometrial cancer with indocyanine green: laparoscopic versus robotic approach

**DOI:** 10.52054/FVVO.13.1.002

**Published:** 2021-03-31

**Authors:** N Bizzarri, S Restaino, S Gueli Alletti, G Monterossi, A Gioè, E La Fera, V Gallotta, A Fagotti, G Scambia, F Fanfani

**Affiliations:** Fondazione Policlinico Universitario A. Gemelli, IRCCS, UOC Ginecologia Oncologica, Dipartimento per la salute della Donna e del Bambino e della Salute Pubblica, Largo Agostino Gemelli 8, 00168, Rome, Italy; Università Cattolica del Sacro Cuore, Largo Francesco Vito 1, 00168, Rome, Italy.

**Keywords:** Endometrial cancer, Sentinel lymph node, Robotic surgery, Laparoscopy, Indocyanine green, minimally invasive surgery

## Abstract

**Background:**

The aims of the present study were to assess bilateral sentinel lymph node (SLN) mapping with laparoscopic versus robotic approach, to assess variables affecting bilateral detection rates and to assess survival difference in patients with no/unilateral, compared to bilateral SLN detection.

**Methods:**

This is a retrospective, single-centre, observational cohort study, including patients with endometrial cancer FIGO stage IA-IVB, treated with minimally invasive primary surgery and undergoing indocyanine green (ICG) injection to detect SLN, between January 2015 and December 2019.

**Results:**

Of the 549 included patients, 286 (52.1%) and 263 (47.9%) underwent the laparoscopic and robotic approach respectively. 387 (70.5%) patients had bilateral SLN mapping, 102 (18.6%) and 60 (10.9%) had unilateral and no mapping, respectively. Patients who underwent the robotic approach were older (median 61 versus 64 years, p=0.046) and had a higher BMI (median 26.0 versus 34.8 kg/m2, p<0.001). No difference in any SLN mapping or in SLN bilateral detection was evident between the laparoscopic or robotic approach (p=0.892 and p=0.507 respectively). Patients with bilateral SLN detection in the entire cohort were younger (p<0.001) and had a better 3-year disease-free survival (DFS) compared to patients with no/unilateral SLN mapping (77.0% versus 66.3%, respectively, p=0.036). No 3-year overall survival (OS) difference was reported (p=0.491).

**Conclusion:**

SLN mapping and bilateral SLN detection with ICG in endometrial cancer was not different in the laparoscopic and robotic approach, even though patients undergoing the robotic approach were older and more obese. Bilateral SLN detection was associated with improved 3-year DFS, but not with 3-year OS, compared to no and unilateral SLN detection.

## Introduction

Endometrial cancer is the most frequent gynaecological cancer in developed countries, with 65,620 estimated new cases and 12,590 estimated deaths in 2020 in the United States (Siegel et al., 2020).

Sentinel lymph node (SLN) mapping is now widely utilised in the staging process for apparent uterine- confined endometrial cancer and this is supported by large literature evidence (Koh et al., 2018; Bodurtha Smith et al., 2017; Rossi et al., 2017). The goal of this strategy is to remove the first tumour draining lymph nodes and to evaluate them by ultra-staging in order to obtain an accurate diagnosis of nodal status with limited surgical morbidity, especially lymphoedema (Geppert et al., 2018). Near-infrared technology and the use of indocyanine green (ICG) recently emerged as the dye of choice for SLN, as it allows a higher bilateral mapping, which is a crucial factor in SLN technique (Papadia et al., 2017; Frumovitz et al., 2018; Rozenholc et al., 2019).

Different studies on SLN in endometrial cancer analysed the efficacy of the laparoscopic (Geppert et al., 2018; Papadia et al., 2017; Papadia et al., 2016) and robotic approach (Casarin et al., 2020; Stephens et al., 2020) in SLN mapping. Robotic surgery has been demonstrated to show peri- operative advantages, especially in morbidly obese endometrial cancer patients, with a significant reduction in conversion rate to laparotomy (Leitao et al., 2016; Corrado et al., 2018). Nevertheless, the increase of body mass index (BMI) has been associated with decreased rate of bilateral SLN detection (Eriksson et al., 2016; Tanner et al., 2015), particularly when blue dye, rather than ICG, is used as a tracer (Sinno et al., 2014). More recently, a significant advantage in terms of overall and bilateral SLN mapping in obese patients with ICG compared with blue dye, was reported (Eriksson et al., 2016).

To the best of our knowledge, there is only one study which specifically compared the robotic versus the laparoscopic approach with regards to SLN detection rate with ICG (Chaowawanit et al., 2020), but this was limited by the low number of patients. The primary aim of this present study was to assess whether there is a difference in bilateral detection rate of SLN in endometrial cancer treated with the laparoscopic versus the robotic approach; secondary aims were to assess variables affecting bilateral detection rates in the entire cohort and to assess survival difference in patients with no and unilateral, compared to bilateral SLN detection.

## Materials and Methods

### 

This is a retrospective, single-centre, observational cohort study, approved by the Institutional Review Board (number DIPUSVSP-26-05-2064). Clinical and pathological data was retrieved from the RedCap ® institutional electronic database. All patients with a histological diagnosis of endometrial cancer, International Federation of Gynecology and Obstetrics – FIGO stage (Pecorelli et al., 2009) IA-IVB, treated with primary surgery between January 2015 and December 2019 at Fondazione Policlinico Agostino Gemelli IRCCS, Rome, Italy, were included.

Only patients who received ICG injection to detect SLN and underwent total hysterectomy and bilateral salpingo-oophorectomy were included. Patients who underwent fertility sparing procedures, neo-adjuvant treatment, in whom hysterectomy was not performed, who had no SLN mapping attempted, or with leiomyosarcoma and endometrial stromal sarcoma histology, were excluded. All patients underwent in a pre-operative pelvic ultrasound scan (US) and a computed tomography (CT) scan of chest-abdomen-pelvis (to exclude distant metastases). Only patients submitted to the minimally invasive surgical approach were included: decision to operate on patients utilising the laparoscopic or robotic approach depended on the patient’s BMI and robotic platform availability. In general, patients with a BMI > 30 kg/m2 were selected for the robotic approach. Only patients with no evidence of enlarged (short axis >10mm) pelvic or para-aortic lymph nodes were submitted to SLN mapping. SLN was detected after 1 ml superficial and deep cervical injections of ICG (diluted with sterile water at 1.25 mg/ml) at 3 and 9 o’clock. ICG injection was performed after docking in the case of robotic surgery. About 10-15 minutes after the cervical injection, the retroperitoneal space was opened, and pelvic lymph nodes were assessed with a near infra-red (NIR) camera (Olympus, Tokyo, Japan in case of laparoscopic or Da Vinci Xi, Intuitive, Sunnyvale, California, US in case of robotic approach). SLN was defined as the ICG-positive lymph node closest to the uterus. Pelvic retroperitoneal spaces were explored with the following order to assess SLN mapping: external iliac, inter-iliac, obturator, common iliac, parametrial and pre-sacral and low para-aortic area.

If no pelvic SLN was detected, the para-aortic area was explored trans-peritoneally and the retroperitoneal para-aortic area was accessed in cases of ICG-positive para-aortic SLN. In cases of apparent early-stage tumours (FIGO stage I-II), if bilateral pelvic SLNs were detected, these were sent to pathology for analysis with ultra-staging or one-step nucleic acid amplification (OSNA) (Fanfani et al., 2018; Monterossi et al., 2019). In case of ultrastaging analysis no further lymph node dissection was performed (Koh et al., 2018), and nodes were sent for final histology. In case of OSNA analysis, the SLN was reported intra-operatively and lymphadenectomy was performed in patients with positive SLNs (for micro-metastasis or macro-metastasis). When SLN was not identified, deep cervical ICG re-injection was performed. In cases with no mapping on a hemi-pelvis, a side- specific pelvic lymphadenectomy was performed (Koh et al., 2018). Moreover, in the first patients of our series, SLN was performed along with pelvic lymphadenectomy as institutional validation of the SLN technique, even in low and intermediate risk patients. Patients with serous histology underwent additional peritoneal staging including infracolic omentectomy and multiple peritoneal biopsies (Koh et al., 2018; Colombo et al., 2016). Adjuvant treatment was administered according to international guidelines (Koh et al., 2018).

### Statistical analysis

Standard descriptive statistics were used to evaluate the distribution of each variable. Continuous variables were reported as median and categorical variables as frequencies or percentages. The distribution of variables between groups was compared with student’s t-test, chi-square test or Fisher’s exact test, as appropriate. Logistic regression analysis was performed to perform univariate and multivariable analyses. Intra- operative complications were graded according to Common Terminology Criteria for Adverse Events (CTCAE) v. 5.0, and post-operative complications were graded according to Clavien-Dindo grading system (Dindo et al., 2004). Disease-free survival (DFS) was defined as the time in months from the date of the surgery to the date of first recurrence, last follow-up or death. Overall survival (OS) was calculated as the time in months from the date of the surgery to the date of the last follow-up or death. OS and DFS were estimated by the Kaplan-Meier method (Kaplan and Meier 1958) and compared by the log-rank test (Mantel 1966). All p-values reported are two-sided and a p-value <0.05 was considered statistically significant. All statistical analyses were performed with SPSS version 26.0 (IBM Corporation 2018, Armonk, NY: IBM Corp.).

## Results

### Entire cohort characteristics

Out of 869 patients who underwent surgery for endometrial cancer in the study period, 549 (63.2%) met the inclusion criteria.

Clinical, surgical and pathological characteristics of included patients are reported in Table I. Median age was 63 years (range, 25-88) and median BMI was 28.8 kg/m2 (range, 16.7-64.1). 286 (52.1%) and 263 (47.9%) patients underwent the laparoscopic and robotic approach respectively. 10 (1.8%) patients required conversion to laparotomy. Reasons for laparotomy conversion were as follow: 5 (0.9%) disease extension beyond uterus, 2 (0.4%) concomitant large ovarian mass, 2 (0.4%) severe adhesions and 1 (0.2%) ureteric injury. Five (0.9%) cases had unexpected histological findings of positive pelvic peritoneum and they were staged as FIGO IVB. Overall, 387 (70.5%) patients had bilateral SLN mapping, while 102 (18.6%) and 60 (10.9%) had unilateral and no mapping, respectively. Systematic pelvic lymphadenectomy (with or without systematic aortic lymphadenectomy) was performed in 214 (39.0%) cases. The median number of harvested pelvic lymph nodes was 11 (range, 3-40) and para-aortic lymph nodes was 9 (range, 1-31) when lymphadenectomy was performed.

**Table I:** Entire cohort characteristics.

Characteristic		N=549, (range, %)
Age (years)		63 (25-88)
BMI (kg/m2)		28.8 (16.7-64.1)
Approach		
	Laparoscopy	286 (52.1)
	Robot	263 (47.9)
Conversion to laparotomy		10 (1.8)
Systematic lymphadenectomy		
	No	335 (61.0)
	Yes	214 (39.0)
SLN detection		
	No	60 (10.9)
	Unilateral	102 (18.6)
	Bilateral	387 (70.5)
SLN analysis		
	Ultrastaging	158 (28.8)
	OSNA	281 (51.2)
	Ultrastaging and OSNA	35 (6.4)
	No ultrastaging/OSNA	75 (13.7)
Median number SLN		2 (1-6)
Intra-operative complications (CTCAE)		
	G1-2	13 (2.4)
	G3-5	0 (0.0)
Post-operative complications (Clavien-Dindo)		
	G1-2	13 (2.4)
	G3-5	4 (0.7)
Histology		
	Endometrioid	457 (83.2)
	Serous	49 (8.9)
	Clear cell	2 (0.4)
	Mixed	35(6.4)
	Carcinosarcoma	4 (0.7)
	Indifferentiated	1 (0.2)
	Not reported	1 (0.2)
Grade		
	1	59 (10.7)
	2	352 (64.1)
	3	121 (22.0)
	Unknown	17 (3.1)
LVSI		
	Negative	365 (66.5)
	Positive	153 (27.9)
	Unknown	31 (5.6)
Maximum tumour diameter (mm)		30 (1-110)
FIGO Stage		
	IA	322 (58.4)
	IB	109 (19.9)
	II	39 (7.1)
	IIIA	6 (1.1)
	IIIB	3 (0.5)
	IIIC1	62 (11.3)
	IIIC2	3 (0.5)
	IVB	5 (0.9)
Lymph node metastasis		67 (12.2)
Survival		
	Recurrences	30 (5.5)
	Deaths	8 (1.4)
Median follow-up, months		11 (0-57)

BMI: body mass index; SLN: sentinel lymph node; OSNA: One- Step Nucleic Acid Amplification; CTCAE: Common Terminology Criteria for Adverse Events; LVSI: lymph-vascular space involvement; FIGO: International Federation of Gynecology and Obstetrics.

Overall, 1019 SLNs were detected and retrieved. Median number of SLNs removed was 2 (range, 1-6) per patient. The most frequent site of SLN mapping was the external iliac in 584 (57.3%), followed by the obturator in 307 (30.1%) and the internal iliac in 64 (6.3%) cases. Six (1.1%) patients had para- aortic mapping; one (0.2%) of these, had isolated para-aortic mapping.

Survival analysis of the entire cohort showed, with a median follow-up of 11 months (range, 0-57), that 30 (5.5%) patients had a recurrence and 8 (1.4%) died of the disease.

Pattern of recurrence was described as follow: 14 (46.7%) vaginal, 9 (30.0%) pelvic or para- aortic lymph nodes, 5 (16.7%) distant (including 1 peritoneal carcinomatosis) and 2 (6.7%) mixed abdominal and distant. Treatment of recurrences was represented by radio-chemotherapy in 16 (53.3%), radical surgery in 8 (26.7%), chemotherapy only in 6 (20.0%) cases.

No 3-year DFS and OS difference was evident when patients undergoing SLN only were compared to patients undergoing SLN and systematic lymphadenectomy (p=0.402 and p=0.267).

### Comparison of laparoscopic and robotic approach

**Figure 1:**
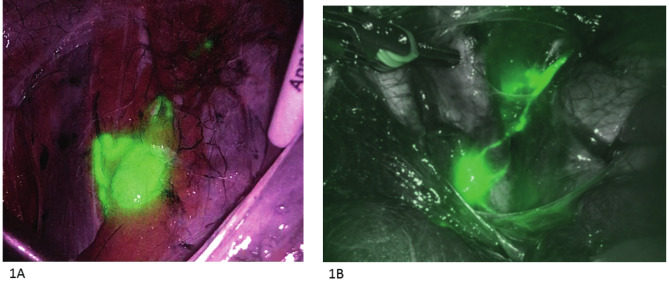
— Examples of laparoscopic (1A) and robotic left external iliac SLN (1B).

Comparison of characteristics of the laparoscopic (286, 52.1%) and the robotic approach (263, 47.9%) are reported in Table II. Patients who underwent the robotic approach were older (median 61 versus 64 years, p=0.046) and had, as expected, a higher BMI (median 26.0 versus 34.8 kg/m2, p<0.001). No difference in conversion to laparotomy was detected (2.8% versus 0.8%, p=0.109). No difference in any SLN mapping or in SLN bilateral detection was evident between the laparoscopic or robotic approach (p=0.892 and p=0.507, respectively). Moreover, there was no difference in median number of SLNs mapped and retrieved between the two approaches (2 in both groups, p=0.650) and in site of SLN mapping (p=0.057). Figure 1A and Figure 1B shows two examples of SLN mapping in laparoscopic and robotic surgery, respectively.

**Table II:** Comparison of characteristics of patients operated with Laparoscopic and Robotic approach.

Characteristic		Laparoscopic N=286, (range, %)	Robotic N=263, (range, %)	p-value
Age (years)		61 (28-88)	64 (25-84)	0.046
BMI (kg/m2)		26.0 (16.7-50.0)	34.8 (18.7-64.1)	<0.001
Conversion to laparotomy		8 (2.8)	2 (0.8)	0.109
Intra-operative complications				0.617
	No	283 (99.0)	260 (98.9)	
	Yes	3 (1.0)	3 (1.1)	
Post-operative complications				0.057
	No	281 (98.3)	251 (95.4)	
	Yes	5 (1.7)	12 (4.6)	
Post-operative complications				0.261
	Grade 1-2	281 (98.3)	251 (95.4)	
	Grade 3-5	5 (1.7)	8 (3.0)	
Histology				0.115
	Endometrioid	229 (80.1)	228 (86.7)	
	Serous	30 (10.5)	19 (7.2)	
	Clear cell	2 (0.7)	0 (0.0)	
	Mixed	20 (7.0)	15 (5.7)	
	Carcinosarcoma	4 (1.4)	0 (0.0)	
	Indifferentiated	1 (0.3)	0 (0.0)	
	Not reported	0 (0.0)	1 (0.4)	
Grade**				0.002
	1	38 (13.9)	21 (8.1)	
	2	162 (59.1)	190 (73.6)	
	3	74 (27.0)	47 (18.2)	
LVSI****				0.441
	Negative	198 (72.0)	167 (68.7)	
	Positive	77 (28.0)	76 (31.3)	
Maximum tumour diameter (mm)		30 (1-110)	30 (3-110)	0.070
FIGO Stage				0.989
	IA	168 (58.7)	154 (58.5)	
	IB	59 (20.6)	50 (19.0)	
	II	19 (6.6)	20 (7.6)	
	IIIA	3 (1.0)	3 (1.1)	
	IIIB	2 (0.7)	1 (0.4)	
	IIIC1	31 (10.8)	31 (11.8)	
	IIIC2	1 (0.3)	2 (0.8)	
	IVB	3 (1.0)	2 (0.8)	
SLN mapping				0.892
	No	32 (11.2)	28 (10.6)	
	Yes	254 (88.8)	235 (89.4)	
SLN detection*				0.507
	Unilateral	56 (22.0)	46 (19.6)	
	Bilateral	198 (78.0)	189 (80.4)	
Number of SLN				0.756
	1	45 (15.7)	35 (13.3)	
	2	128 (44.8)	114 (43.3)	
	4	56 (19.6)	52 (19.8)	
	6	2 (0.7)	1 (0.4)	
Median number of SLN		2 (1-6)	2 (1-6)	0.650
Site of mapping of first SLN****				0.057
	Obturator	181 (33.5)	126 (26.3)	
	Internal iliac	34 (6.3)	30 (6.3)	
	External iliac	297 (55.0)	287 (59.9)	
	Common iliac	18 (3.3)	30 (6.3)	
	Pre-sacral	6 (1.1)	4 (0.8)	
	Para-aortic	3 (0.5)	2 (0.4)	
	Para-aortic (isolated)	1 (0.2)	0 (0.0)	
SLN metastasis				0.213
	No	254 (88.8)	236 (89.7)	
	ITC	6 (2.1)	6 (2.3)	
	Micro	21 (7.3)	11 (4.2)	
	Macro	5 (1.7)	10 (3.8)	

BMI: body mass index; LVSI: lymph-vascular space involvement; FIGO: International Federation of Gynecology and Obstetrics; SLN: sentinel lymph node.; *32 LPS and 28 Robotic did not map; ** 17 unknown *** 31 unknown; **** data shows lymph nodes in 433 cases (60 no mapping excluded; data not reported in 56); total number of SLNs retrieved: 1019.

### Comparison of no/unilateral and bilateral SLN detection

Analysis of variables associated with bilateral SLN detection, compared with no/unilateral SLN detection within the entire cohort, are reported in Table III. Age was the only patient-related characteristic which was associated with bilateral SLN detection: patients with bilateral SLN detection were younger than patients with no/unilateral SLN detection (median, 61 versus 66 years, respectively; p<0.001). When we analysed surgery-related variables, after dividing the study period in two (learning period until 15/06/2017 and experienced period after 15/06/2017), we noted that bilateral SLN detection was more frequent in the experienced period: 26/47 (55.3%) versus 361/502 (71.9%) bilateral SLN detection in the learning and experienced period respectively (p=0.028). However, no bilateral SLN detection difference was evident when the two learning periods were stratified according to the surgical approach, laparoscopy versus robotic (p=0.758 and p=0.427, for the first and second period, respectively). Age < 65 years and an experienced period of surgery were the only variables related to bilateral SLN detection at multivariable analysis (Table IV).

**Table III:** Factors associated with bilateral detection in the entire cohort.

Factors associated with bilateral detection in the entire cohort		No/Unilateral Mapping (N=162), (range, %)	Bilateral Mapping (N=387), (range, %)	p-value
Patient/Tumour-related variables				
Age (years)		66 (40-88)	61 (25-87)	<0.001
BMI (kg/m2)		30 (18-55)	29 (17-64)	0.222
Obesity (BMI>30kg/m2)				0.157
	No	83 (51.2)	225 (58.1)	
	Yes	79 (48.8)	162 (41.9)	
Prior pelvic surgery				0.496
	No	99 (61.1)	248 (64.4)	
	Yes	63 (38.9)	137 (35.6)	
Previous vaginal delivery				0.702
	No	67 (41.4)	152 (39.3)	
	Yes	95 (58.6)	235 (60.7)	
Number of vaginal deliveries		1.5 (0-6)	1 (0-7)	0.877
Caesarean Section				0.123
	No	124 (76.5)	319 (82.4)	
	Yes	38 (23.5)	69 (17.5)	
Number of caesarean section		0 (0-3)	0 (0-4)	0.078
Histology				0.076
	Endometrioid	128 (79.0)	331 (85.5)	
	Non-endometrioid	34 (21.0)	56 (14.5)	
Grade*				0.579
	1	16 (10.3)	43 (11.4)	
	2	100 (64.1)	252 (67.0)	
	3	40 (25.6)	81 (21.5)	
	Unknown			
LVSI**				
	Negative	103 (68.2)	262 (71.4)	
	Positive	48 (31.8)	105 (28.6)	
	Maximum tumour diameter (mm)	30 (1-100)	32 (1.5-110)	0.675
Cervical stroma invasion				0.071
	No	138 (85.2)	351 (90.7)	
	Yes	24 (14.8)	36 (9.3)	
Tumour diameter				0.967
	< 20mm	35 (21.6)	83 (21.4)	
	≥ 20mm	127 (78.4)	304 (78.6)	
FIGO Stage				0.071
	I-II	139 (85.8)	331 (85.5)	
	III-IV	23 (14.2)	56 (14.5)	
Lymph node metastasis				
	No	142 (87.7)	340 (87.8)	0.948
	Yes	20 (12.3)	47 (12.1)	
Adjuvant treatment				
	No	70 (43.2)	(170 (43.9)	0.925
	Yes	92 (56.8)	217 (56.1)	
Surgery-related variables				
Period of surgery				0.028
	Learning period (01.2015/06.2017)	21 (13.0)	26 (6.7)	
	Experienced period (06.2017/12.2019)	141 (87.0)	361 (93.3)	
Approach				0.513
	Laparoscopy	88 (54.3)	198 (51.2)	
	Robot	74 (45.7)	189 (48.8)	
Intra-operative complications (CTCAE)				0.676
	No	161 (99.4)	382 (98.7)	
	Yes	1 (0.6)	5 (1.3)	
Post-operative complications (Clavien-Dindo)				0.012
	No	152 (93.8)	380 (98.2)	
	Yes	10 (6.2)	7 (1.8)	
Post-operative complications				0.682
	Grade 1-2	8 (80.0)	5 (71.4)	
	Grade 3-5	2 (20.0)	2 (28.6)	
Site of mapping***				0.352
	Pelvic	102 (100.0)	381 (98.4)	
	Para-aortic	0 (0.0)	6 (1.6)	
SLN metastasis				0.022
	No	153 (94.4)	337 (87.1)	
	ITC	1 (0.6)	11 (2.8)	
	Micro	3 (1.9)	29 (7.5)	
	Macro	5 (3.1)	10 (2.6)	

* 17 unknown; ** 31 unknown; *** data on 489 cases (no mapping excluded)

BMI: body mass index; LVSI: lymph-vascular space involvement; FIGO: International Federation of Gynecology and Obstetrics; SLN: sentinel lymph node; CTCAE: Common Terminology Criteria for Adverse Events.

**Table IV:** Univariate and multivariate logistic regression analysis analysing factors associated with bilateral detection in the entire cohort.

		Univariate analysis		Multivariable analysis	
Characteristic*		Odds Ratio (95% CI)	p-value	Odds Ratio (95% CI)	p-value
Age		0.483 (0.333-0.701)	< 0.001	0.506 (0.346-0.741)	< 0.001
	< 65 years				
	≥ 65 years				
Period of surgery		0.484 (0.264-0.887)	0.019	0.464 (0.250-0.862)	0.015
	Learning period				
	Experienced period				
Number of previous caesarean sections		0.801 (0.636-1.011)	0.061		
	0-1				
	> 1				
Histology		1.629 (1.019-2.604)	0.042	1.434 (0.883-2.331)	0.145
	Endometrioid				
	Non-endometrioid				
Cervical stroma involvement		0.590 (0.339-1.025)	0.061		
	No				
	Yes				

*Variables with a p-value < 0.10 at Fisher’s/Chi square test in Table III, were included in the logistic regression analysis.

There was no difference in intra-operative complication rate between patients who had bilateral and patients who did not have bilateral SLN mapping. Post-operative complications were more frequent in patients who did not have bilateral mapping (6.2% versus 1.8%, p=0.012). However, no difference in severe post-operative complications was recorded (p=0.682). Lastly, patients with bilateral SLN detection were found to have higher numbers of SLN metastases: in particular they had a higher rate of isolated tumour cells (ITCs) and micro-metastases (p=0.022).

**Figure 2:**
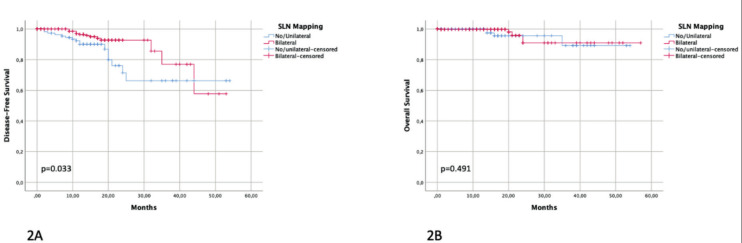
— Disease-free survival (2A) and overall survival (2B) in no/unilateral versus bilateral SLN mapping.

Survival comparison demonstrated that patients with bilateral SLN mapping had a better 3-year DFS compared to patients with no/unilateral SLN mapping (77.0% versus 66.3%, respectively, p=0.036) (Figure 2A). No difference in 3-year OS between the two groups was reported (90.9% versus 89.2%, p=0.491) (Figure 2B).

## Discussion

With the present study, we demonstrated that endometrial cancer patients operated using the robotic or laparoscopic approach had no difference in SLN mapping and bilateral detection rate, despite the signifi cantly higher BMI of patients submitted to robotic surgery. This is in contrast with previous studies, which correlated the higher BMI with no or unilateral SLN mapping (Eriksson et al., 2016; Tanner et al., 2015). Moreover, this is in contrast with a very recent study that found a higher overall detection rate using the laparoscopic approach, compared to robotic (with no difference in bilateral detection rate) (Chaowawanit et al., 2020). Nevertheless, other studies reported that BMI was not a clinical characteristic which infl uenced the rate of bilateral SLN detection (Stephens et al., 2020; Tortorella et al., 2019; Body et al., 2018). Furthermore, our results further support the use of robotic surgery in obese and morbidly obese women, as previously reported by other authors (Leitao et al., 2016; Corrado et al., 2018).

The relevance of bilateral SLN detection is explained by the fact that SLN can be analysed with thorough examination, avoiding systematic lymphadenectomy related morbidity (Geppert et al., 2018).

When we analysed the factors that potentially infl uence bilateral SLN detection in the entire cohort, we found that age was the only patient-related signifi cant variable, confi rmed at multivariable analysis. Most of the studies previously reported other variables signifi cantly related with bilateral SLN mapping, such as use of ICG, low BMI, no clinically enlarged lymph nodes, early FIGO stage, no lysis of adhesions at the beginning of surgery and surgeon’s experience in SLN biopsy (Eriksson et al., 2016; Tanner et al., 2015; Tortorella et al., 2019; Body et al., 2018; Harold et al., 2019; Sozzi et al., 2020). The relationship between increased age and lymphatic dysfunction has been described: in particular, cell senescence, impaired contractile function and decrease of nitric oxide in aged lymphatic collectors, may lead to poor drainage of lymph, causing bilateral mapping failure (Shang et al., 2019).

In our study, we confi rmed that the learning curve of ICG injection and SLN mapping, signifi cantly affected the rate of bilateral SLN detection, by observing a significantly higher bilateral SLN detection in the second period of our series. As expected, patients with no/unilateral SLN detection had signifi cantly higher post-operative complications, most probably due to uni- or bilateral pelvic lymphadenectomy performed. Nevertheless, no difference in severe post-operative complications was found between patients with or without bilateral SLN detection.

As previously reported (Bogani et al., 2019), a higher rate of low-volume metastases (ITC and micro- metastasis) was observed in patients with successful bilateral SLN mapping. We could assume that this is a consequence of the more accurate analysis of the SLN, rather than single section analysis of multiple lymph nodes in lymphadenectomy specimens by standard hematoxylin and eosin (H&E).

At survival analysis, a signifi cant better 3-year DFS in the group of patients with bilateral SLN detection compared to those with no/unilateral SLN detection was observed (p=0.033). On the contrary, no signifi cant differences in terms of OS between the two groups was observed. Therefore, SLN can be interpreted as a more accurate tool to detect positive lymph nodes, with consequent tailored adjuvant treatment. Nevertheless, the lack of OS impact could indicate that patients with recurrent disease can be successfully treated at relapse (Connor et al., 2018; Legge et al., 2020).

We have to acknowledge the retrospective nature, a possible selection bias to the surgical approach and the short median follow up, as main limitations of the present study. On the other hand, we have to recognise the large number of patients submitted to ICG SLN from a single institution and the fact that this is one of the fi rst studies comparing performance of the laparoscopic and robotic approach in SLN mapping.

## Conclusion

SLN mapping and bilateral detection rates in endometrial cancer were no different between the laparoscopic or robotic approach, even though patients undergoing the robotic approach were older and more obese. Younger age affected the bilateral SLN detection rate in the entire cohort. Bilateral SLN detection was associated with improved 3-year DFS, but not with 3-year OS, compared to patients with no and unilateral SLN detection.

## References

[1] Bodurtha Smith AJ, Fader AN (2017). Sentinel lymph node assessment in endometrial cancer: a systematic review and meta-analysis.. Am J Obstet Gynecol.

[2] Body N, Grégoire J, Renaud MC (2018). Tips and tricks to improve sentinel lymph node mapping with Indocyanin green in endometrial cancer.. Gynecol Oncol.

[3] Bogani G, Murgia F, Ditto A (2019). Sentinel node mapping vs. lymphadenectomy in endometrial cancer: A systematic review and meta-analysis. Gynecol Oncol.

[4] Casarin J, Multinu F, Tortorella L (2020). Sentinel lymph node biopsy for robotic-assisted endometrial cancer staging: further improvement of perioperative outcomes.. Int J Gynecol Cancer.

[5] Chaowawanit W, Campbell V, Wilson E (2020). Comparison between laparoscopic and robotic surgery for sentinel lymph node mapping in endometrial cancer using indocyanine green and near infra-red fluorescence imaging.. J Obstet Gynaecol.

[6] Colombo N, Creutzberg C, Amant F (2016). ESMO-ESGO-ESTRO Consensus Conference on Endometrial Cancer: diagnosis, treatment and follow-up.. Ann Oncol.

[7] Common Terminology Criteria for Adverse Events (CTCAE) v5.0.

[8] Connor EV, Rose PG (2018). Management Strategies for Recurrent Endometrial Cancer.. Expert Rev Anticancer Ther.

[9] Corrado G, Vizza E, Cela V (2018). Laparoscopic versus robotic hysterectomy in obese and extremely obese patients with endometrial cancer: A multi-institutional analysis.. Eur J Surg Oncol.

[10] Dindo D, Demartines N, Clavien PA (2004). Classification of surgical complications: a new proposal with evaluation in a cohort of 6336 patients and results of a survey.. Ann Surg.

[11] Eriksson AG, Montovano M, Beavis A (2016). Impact of Obesity on Sentinel Lymph Node Mapping in Patients with Newly Diagnosed Uterine Cancer Undergoing Robotic Surgery.. Ann Surg Oncol.

[12] Fanfani F, Monterossi G, Ghizzoni V (2018). One-Step Nucleic Acid Amplification (OSNA): A fast molecular test based on CK19 mRNA concentration for assessment of lymph-nodes metastases in early stage endometrial cancer.. PLoS One.

[13] Frumovitz M, Plante M, Lee PS (2018). Near-infrared fluorescence for detection of sentinel lymph nodes in women with cervical and uterine cancers (FILM): a randomised, phase 3, multicentre, non-inferiority trial. Lancet Oncol.

[14] Geppert B, Lönnerfors C, Bollino M (2018). Sentinel lymph node biopsy in endometrial cancer-Feasibility, safety and lymphatic complications.. Gynecol Oncol.

[15] Harold JA, Uyar D, Rader JS (2019). Adipose-only sentinel lymph nodes: a finding during the adaptation of a sentinel lymph node mapping algorithm with indocyanine green in women with endometrial cancer.. Int J Gynecol Cancer.

[16] Kaplan EL, Meier P (1958). Nonparametric estimation from incomplete observation.. J Am Stat Assoc.

[17] Koh WJ, Abu-Rustum NR, Bean S (2018). Uterine Neoplasms, Version 1.2018, NCCN Clinical Practice Guidelines in Oncology. J Natl Compr Canc Netw.

[18] Legge F, Restaino S, Leone L (2020). Clinical outcome of recurrent endometrial cancer: analysis of post-relapse survival by pattern of recurrence and secondary treatment.. Int J Gynecol Cancer.

[19] Leitao MM, Narain WR, Boccamazzo D (2016). Impact of robotic platforms on surgical approach and costs in the management of morbidly obese patients with newly diagnosed uterine cancer.. Ann Surg Oncol.

[20] Mantel N (1966). Evaluation of survival data and two new rank order statistics arising in its consideration. Cancer Chem Rep.

[21] Monterossi G, Buca D, Dinoi G (2019). Intra-operative assessment of sentinel lymph node status by one-step nucleic acid amplification assay (OSNA) in early endometrial cancer: a prospective study.. Int J Gynecol Cancer.

[22] Papadia A, Imboden S, Siegenthaler F (2016). Laparoscopic indocyanine green sentinel lymph node mapping in endometrial cancer.. Ann Surg Oncol.

[23] Papadia A, Zapardiel I, Bussi B (2017). Sentinel lymph node mapping in patients with stage I endometrial carcinoma: a focus on bilateral mapping identification by comparing radiotracer Tc99m with blue dye versus indocyanine green fluorescent dye.. J Cancer Res Clin Oncol.

[24] Pecorelli S (2009). Revised FIGO staging for carcinoma of the vulva, cervix, and endometrium.. Int J Gynaecol Obstet.

[25] Rossi EC, Kowalski LD, Scalici J (2017). A comparison of sentinel lymph node biopsy to lymphadenectomy for endometrial cancer staging (FIRES trial): a multicentre, prospective, cohort study.. Lancet Oncol.

[26] Rozenholc A, Samouelian V, Warkus T (2019). Green versus blue: Randomized controlled trial comparing indocyanine green with methylene blue for sentinel lymph node detection in endometrial cancer.. Gynecol Oncol.

[27] Shang T, Liang J, Kapron CM (2019). Pathophysiology of aged lymphatic vessels.. Aging (Albany NY).

[28] Siegel RL, Miller KD, Jemal A (2020). Cancer statistics, 2020.. CA Cancer J Clin.

[29] Sinno AK, Fader AN, Roche K (2014). A comparison of colorimetric versus fluorometric sentinel lymph node mapping during robotic surgery for endometrial cancer.. Gynecol Oncol.

[30] Sozzi G, Fanfani F, Berretta R (2020). Laparoscopic sentinel node mapping with intracervical indocyanine green injection for endometrial cancer: the SENTIFAIL study - a multicentric analysis of predictors of failed mapping.. Int J Gynecol Cancer.

[31] Stephens AJ, Kennard JA, Fitzsimmons CK (2020). Robotic sentinel lymph node (SLN) mapping in endometrial cancer: SLN symmetry and implications of mapping failure.. Int J Gynecol Cancer.

[32] Tanner EJ, Sinno AK, Stone RL (2015). Factors associated with successful bilateral sentinel lymph node mapping in endometrial cancer.. Gynecol Oncol.

[33] Tortorella L, Casarin J, Multinu F (2019). Sentinel lymph node biopsy with cervical injection of indocyanine green in apparent early-stage endometrial cancer: predictors of unsuccessful mapping.. Gynecol Oncol.

